# The characteristics of intestinal microbiota in patients with type 2 diabetes and the correlation with the percentage of T-helper cells

**DOI:** 10.3389/fmicb.2024.1443743

**Published:** 2024-09-27

**Authors:** Fan Yang, Jinyan Li, Longqin Wei, Shenghua Qin, Qingfeng Shi, Siyan Lu, Shuyuan Chu

**Affiliations:** ^1^Department of Endocrinology, Guilin People's Hospital, Guilin, China; ^2^Research Service Department, Guilin People's Hospital, Guilin, China; ^3^Department of Endocrinology, Affiliated Hospital of Guilin Medical University, Guilin, China; ^4^Medical Department, The Reproductive Hospital of Guangxi Zhuang Autonomous Region, Nanning, China; ^5^Health Management Center, Guilin People's Hospital, Guilin, China; ^6^Laboratory Department, Guilin People's Hospital, Guilin, China; ^7^Rheumatology and Immunology Department, The Second Affiliated Hospital of Guilin Medical University, Guilin, China; ^8^Laboratory of Respiratory Disease, Affiliated Hospital of Guilin Medical University, Guilin, China; ^9^Guangxi Clinical Research Center for Diabetes and Metabolic Diseases, The Second Affiliated Hospital of Guilin Medical University, Guilin, China

**Keywords:** type 2 diabetes (T2D), intestinal microbiota, T-helper cell, immune response, interaction

## Abstract

**Background:**

Type 2 diabetes (T2D) is related to intestinal microflora changes and immune inflammation. We aimed to investigate the pattern of intestinal flora-systematic T helper (Th) cell linkage in T2D patients.

**Methods:**

Participants with T2D diagnosed by physicians and healthy controls were enrolled in the study. The Th1, Th2, and Th17 cells from the peripheral blood were assessed by flow cytometry. The feces were collected. The V3–V4 variable region of 16S rRNA was sequenced and analyzed using bioinformatics. Principal coordinate analysis (PCoA) and non-metric multidimensional scaling (NMDS) analysis were performed to assess the beta diversity. The linear discriminant analysis (LDA) effect size (LEfSe) method was applied to identify amicrobial taxon specific to T2D. The Phylogenetic Investigation of Communities by Reconstruction of Unobserved States (PICRUSt) was conducted to identify the metabolic pathways. A network analysis was conducted by constructing a co-occurrence network.

**Results:**

The percentages of the Th1 and Th17 cells in the peripheral blood were higher in patients with T2D than in controls. Among the top 30 genera of the intestinal microbiota, the levels of *Lachnospiraceae*_NK4A136_group, *Ruminococcaceae*_UCG002, and *Eubacterium_hallii*_group were lower in the patients with T2D than in controls. In the LEfSe analysis, it was observed that the *Lachnospiraceae* and *Ruminococcaceae* families were significantly different between patients with T2D and controls. Moreover, the Th1/Th2 ratio was positively correlated with the abundance of the *Lachnoclostridium* and *Ruminococcus_torques*_group genera. In the network analysis, the Th1/Th2 ratio, *Ruminococcaceae*_UCG-002, and *Lachnospiraceae*_NK4A136_group were the important nodes.

**Conclusion:**

This study provided a preliminary picture of the crosstalk between the intestinal microbiome and systematic Th cells in patients with T2D. The findings of the study suggested that the network relationship among the intestinal microbiota, metabolites, and CD4+T lymphocyte immunity was unbalanced in the patients with T2D, which might have promoted the development of T2D. This presents a therapeutic opportunity to modulate gut immune reaction and then chronic inflammation by manipulating microbiome-specific Th-cell response.

## 1 Introduction

Type 2 diabetes (T2D) is a progressive metabolic disease characterized by pancreatic β-cell dysfunction and peripheral insulin resistance, leading to defects in glucose metabolism and chronic low-grade inflammation (Zhou et al., 2022). T2D accounts for more than 90% of all diabetes patients (Zheng et al., 2018). In recent years, the intestinal microbiota has been demonstrated to be altered in diabetic populations and animal models, implying that it may be an important participant in the pathogenesis of T2D (Zhou et al., 2022).

The intestinal microbiota has the ability to alter host glucose homeostasis through multiple mechanisms, which include the following: producing metabolites during fermentation and their resulting secondary effects; activating inflammatory cascades, which leads to the release of cytokines; disrupting the permeability of the intestinal mucosal barrier, which allows the influx of toxins; and directly activating signaling through incretin secretion (Cunningham et al., 2021). Among these mechanisms, the decreasing beneficial microflora and increasing pathogenic microflora could cause low-grade inflammation in the gut, which can lead to insulin resistance and T2DM (Aydin et al., 2023).

In T2D, CD4+ T cells participate in the pathology of insulin resistance, which relates to chronic inflammation (Zhou et al., 2018). The differentiation of CD4+ T helper (Th) cells, including Th1, Th2, and Th17, was found to be abnormal in the peripheral blood of patients with T2D, which could have been induced by glycemic variability (Zhou et al., 2018; Sun et al., 2024). The abnormal differentiation of CD4+ Th cell subpopulations may impair β-cell function, aggravate insulin resistance, and contribute to the development of T2D through producing cytokines and participating in chronic and low-grade inflammation (Aydin et al., 2023; Drenthen et al., 2024; Han et al., 2024; Valentine and Nikolajczyk, 2024). Thus, CD4+ Th subsets play key roles in the pathogenesis of T2D.

The microbiota could mediate Th-cell differentiation and homeostasis in pathogenic conditions through the gut microbiota–T cell axis (Shim et al., 2023). On the one hand, in a diabetic model, it has been demonstrated that high-fat diet (HFD)-induced gut microbiota causes a reduction in intestinal Th17 by impairing the ability of intestinal antigen-presenting cells to induce functional Th17 cells (Garidou et al., 2015; Céline et al., 2016). On the other hand, in another study, the Th17 response in the small intestine of HFD-induced T2D mice was found to be important to shape a healthy gut microbiota and to maintain the gut barrier integrity (Carlos et al., 2020). However, recent findings about the intestinal flora–Th cells axis in T2D have mostly focused on the intestinal Th cells based on a murine model. The crosstalk between the intestinal microbiome and systematic Th subsets in patients with T2D remains unclear. Therefore, in this study, we aimed to investigate the pattern of intestinal flora–systematic Th cells linkage in patients with T2D. We assessed the intestinal microbiota and Th cells from the peripheral blood in patients with T2D and further explored the relationship between the intestinal microbiota and the percentage of Th cells in this study.

## 2 Methods

### 2.1 Study participants

From June 2020 to December 2020, participants with T2D diagnosed by physicians according to the guidelines from the American Diabetes Association (2020) between the ages of 30 and 85 years were recruited in the case group from the Affiliated Hospital of Guilin Medical University, Guilin, China. The inclusion criteria for the participants in the case group included the following: (1) T2D was diagnosed for the first time, (2) the period of T2D symptoms was < 1 year, (3) the participants did not accept the administration of medicine or nutrition for T2D, and (4) the participants had a common diet in their routine. The participants in the case group were excluded if they had any other type of diabetes other than T2D, had autoimmune disorders, asthma, chronic obstructive pulmonary disease, malignant tumor, HIV infection, and gastrointestinal diseases within the last two weeks, had accepted treatment with glucocorticoid within the last three years, or had accepted antibiotics or microecologics within the last two weeks. The controls were healthy participants in the same age range from the health management center of the same hospital.

The peripheral blood and feces samples were collected from each participant in the morning after they were recruited for the study on an empty stomach. Fresh feces of 3–5 g were collected from each participant and stored at −80°C.

The study protocol was approved by the Institutional Review Board at the Affiliated Hospital of Guilin Medical University (No. 2021GLMU1AYJS024) and conformed to the Declaration of Helsinki. Written informed consent was obtained from each participant.

### 2.2 Flow cytometry

The immunophenotype of CD4+T subtypes was investigated by flow cytometry from the peripheral blood mononuclear cells (PBMCs). Before staining, the samples (4 × 10^6^ cells/ml) with 500 μl in a 24-well plate were stimulated with 2-μl Cell Activation Cocktail (without Brefeldin A) and 1-μl Brefeldin A in each well at 37°C and 5% CO2 for 6 h. Th1 lymphocytes were identified as CD4+ (FITC) and IFN-γ (PerCP/Cyanine5.5); Th2 lymphocytes were identified as CD4+ (FITC) and IL-4 (APC); and Th17 lymphocytes were identified as CD4+ (FITC) and IL-17A+ (PE). All antibodies were purchased from Biolegend, San Diego, USA. All flow cytometry experiments were performed using the CytoFLex flow cytometer and CytExpert software (Beckman Coulter).

### 2.3 Intestinal microbiota determination

The methods were similar to the methods described in a previous report (Su et al., 2021). In brief, total bacterial DNA was extracted from the fecal samples using a magnetic fecal DNA extraction kit, and the quality of the extracted DNA was identified by employing agarose gel electrophoresis. The V3–V4 variable region of the 16S rRNA was amplified using a polymerase chain reaction (PCR). The primers with a barcode were as follows: 343F, 5′-TACGGRAGGCAGCAG-3′ and 798R, 5′-AGGGTATCTAATCCT-3′. The PCR products were detected using 2% agarose gel electrophoresis and then montaged and sequenced using the Trimmomatic and FLASH software. VSEARCH software was used to classify operational taxonomic unit (OTU) representative sequences with a 97% similarity level. The community composition of the samples was analyzed at all levels (kingdom, phylum, class, order, family, genus, and species) and clustered into OTUs before being compared with the data in the Greengenes database. The species classification information was annotated using the QIIME software. The OTUs (species richness) and diversity (the Chao and Shannon diversity indices) indices were analyzed.

### 2.4 Statistical and bioinformatics analysis

The data were expressed as means ± standard deviation (SD). The Th1, Th2, Th17, and Th1/Th2 ratios were expressed as median (range). Significant differences were evaluated using an independent samples *t*-test or the chi-square test. Correlation coefficients were calculated using the Spearman correlation coefficient method. A *p*-value of < 0.05 was considered to be statistically significant. All statistical tests were performed using SPSS 21.0 (IBM SPSS Inc.).

Principal coordinate analysis (PCoA) and non-metric multidimensional scaling (NMDS) analysis were used to assess the beta diversity. The linear discriminant analysis (LDA) effect size (LEfSe) method was applied to identify a microbial taxon specific to T2D. The Phylogenetic Investigation of Communities by Reconstruction of Unobserved States (PICRUSt) was conducted to identify the metabolic pathways. A network analysis was performed by constructing a co-occurrence network. Bioinformatics analyses were performed using R version 4.3.2.

## 3 Results

### 3.1 Participants' baseline

A total of 36 participants were included in the case group, and 20 healthy controls were included in the control group. The baseline is shown in Table 1. In both groups, there were more male participants than female ones. The age and BMI were similar in both groups. In the peripheral blood, the neutrophil count, lymphocyte count, monocyte count, levels of creatinine, serum uric acid, triglyceride, cholesterol, apolipoprotein A, and lipoprotein (a) were all similar in the two groups. Moreover, the HbA1C, fasting blood glucose, low-density lipoprotein, and apolipoprotein B levels in the peripheral blood were higher in the case group than in the control group. In contrast, the high-density lipoprotein level in the peripheral blood was lower in the case group than in the control group.

**Table 1:** Participants' characteristics.

**Variables**	**Case group (*N* = 36)**	**Control group (*N* = 20)**	***p*-value**
Age (years)	53.1 ± 10.8	56.8 ± 6.8	0.177
Sex (male)	25 (69.4%)	13 (65.0%)	0.966
BMI (kg/m^2^)	24.6 ± 3.8	23.9 ± 4.9	0.560
Lymphocyte count (109/L)	2.09 ± 0.56	1.85 ± 0.43	0.093
Monocyte count (109/L)	0.52 ± 0.14	0.48 ± 0.16	0.325
Neutrophil count (109/L)	4.14 ± 1.41	3.8 ± 1.13	0.362
FPG (mmol/L)	11.96 ± 4.5	5.03 ± 0.38	< 0.001
HbA1C (%)	10.72 ± 2.71	5.39 ± 0.3	< 0.001
TG (mmol/L)	2.88 ± 4.09	1.84 ± 1.62	0.360
CHOL (mmol/L)	5 ± 1.83	4.37 ± 0.66	0.220
LDLc (mmol/L)	3.13 ± 0.95	2.52 ± 0.55	0.028
HDLc (mmol/L)	1.03 ± 0.3	1.24 ± 0.41	0.050
Apolipoprotein A (g/L)	1.1 ± 0.11	1.12 ± 0.09	0.430
Apolipoprotein B (g/L)	0.98 ± 0.18	0.84 ± 0.15	0.015
Lipoprotein (a) (mg/dL)	18.41 ± 26.55	36.61 ± 41.74	0.148
Creatinine (μmol/L)	71.51 ± 17.23	73.83 ± 14.47	0.612
Uric acid (μmol/L)	320.08 ± 102.81	358.67 ± 81.46	0.155
CD4+T cell (%)	44.274 ± 18.529	45.233 ± 21.373	0.824
Th1 (%)	3.34 (0.57–31.82)	1.18 (0.17–30.00)	0.012
Th2 (%)	1.09 (0.18–18.18)	0.88 (0.11–19.57)	0.726
Th17 (%)	3.41 (0.63–31.15)	1.05 (0.14–11.06)	0.039
Th1/Th2 ratio	2.46 (0.08–69.17)	1.77 (0.04–31.25)	0.108

FPG, fasting blood glucose; TG, triglycerides; CHOL, cholesterol; LDLc, low-density lipoprotein cholesterol; HDLc, high-density lipoprotein cholesterol.

### 3.2 CD4+T cell subtypes

**Figure 1:**
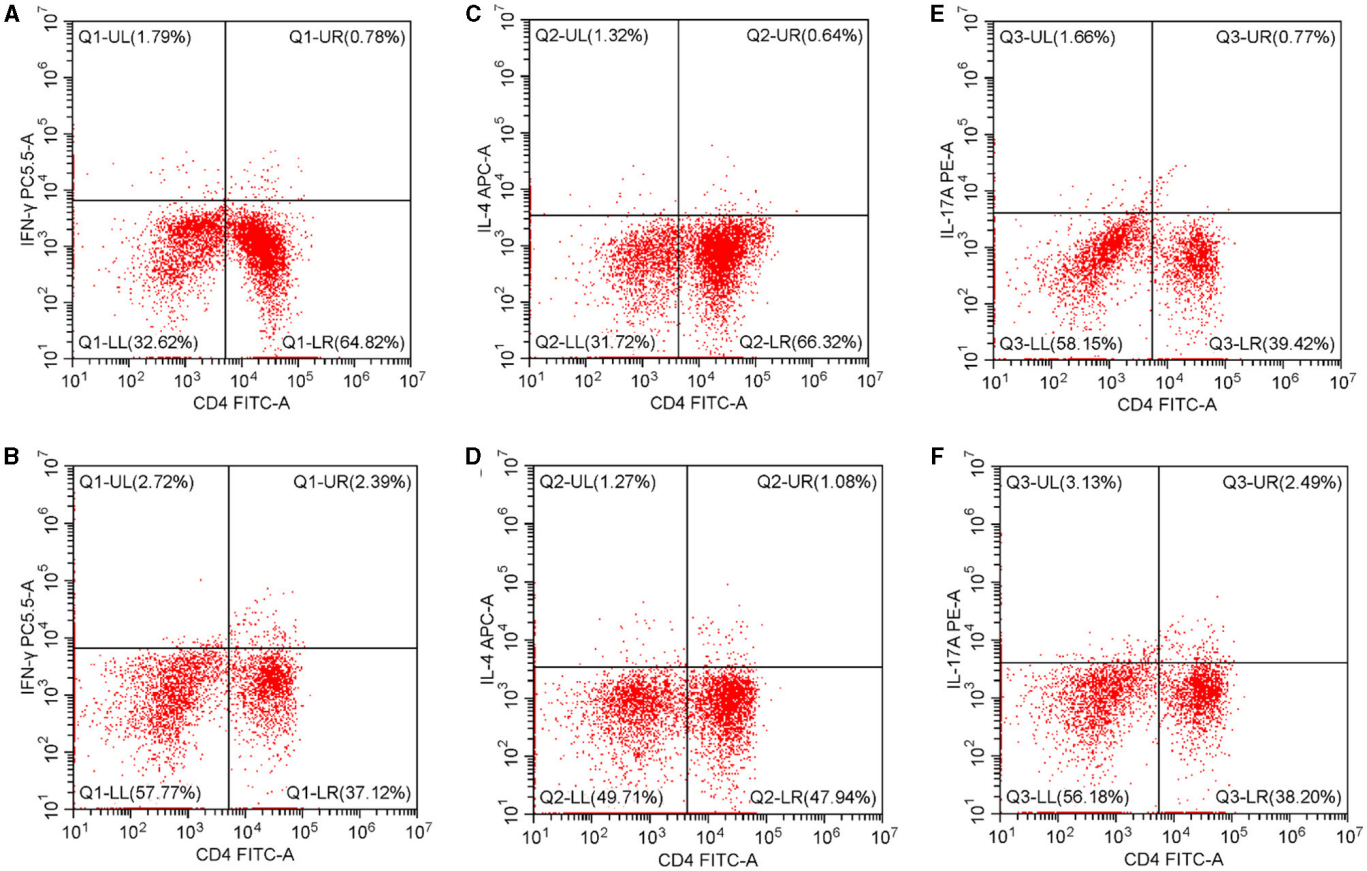
Th1, Th2, and Th17 percentages in the peripheral blood. **(A)** The Th1 percentage in the controls. **(B)** The Th1 percentage in T2D patients. **(C)** The Th2 percentage in controls. **(D)** The Th2 percentage in T2D patients. **(E)** The Th17 percentage in controls. **(F)** The Th17 percentage in T2D patients. T2D, type 2 diabetes.

As Figure 1 illustrates, in the peripheral blood, the percentages of Th1 and Th17 were higher in the case group than in the control group [Th1: 3.34 (0.57–31.82)% vs. 1.18 (0.17–30.00)%, *p* < 0.05; Th17: 3.41 (0.63–31.15)% vs. 1.05 (0.14–11.06)%, *p* < 0.05]. The Th2 percentage and Th1/Th2 ratio were similar in both groups [Th2: 1.09 (0.18–18.18)% vs. 0.88 (0.11–19.57)%, *p* > 0.05; Th1/Th2 ratio: 2.46 (0.08–69.17) vs. 1.77 (0.04–31.25), *p* > 0.05].

### 3.3 Intestinal microbiota in the genus level

**Figure 2:**
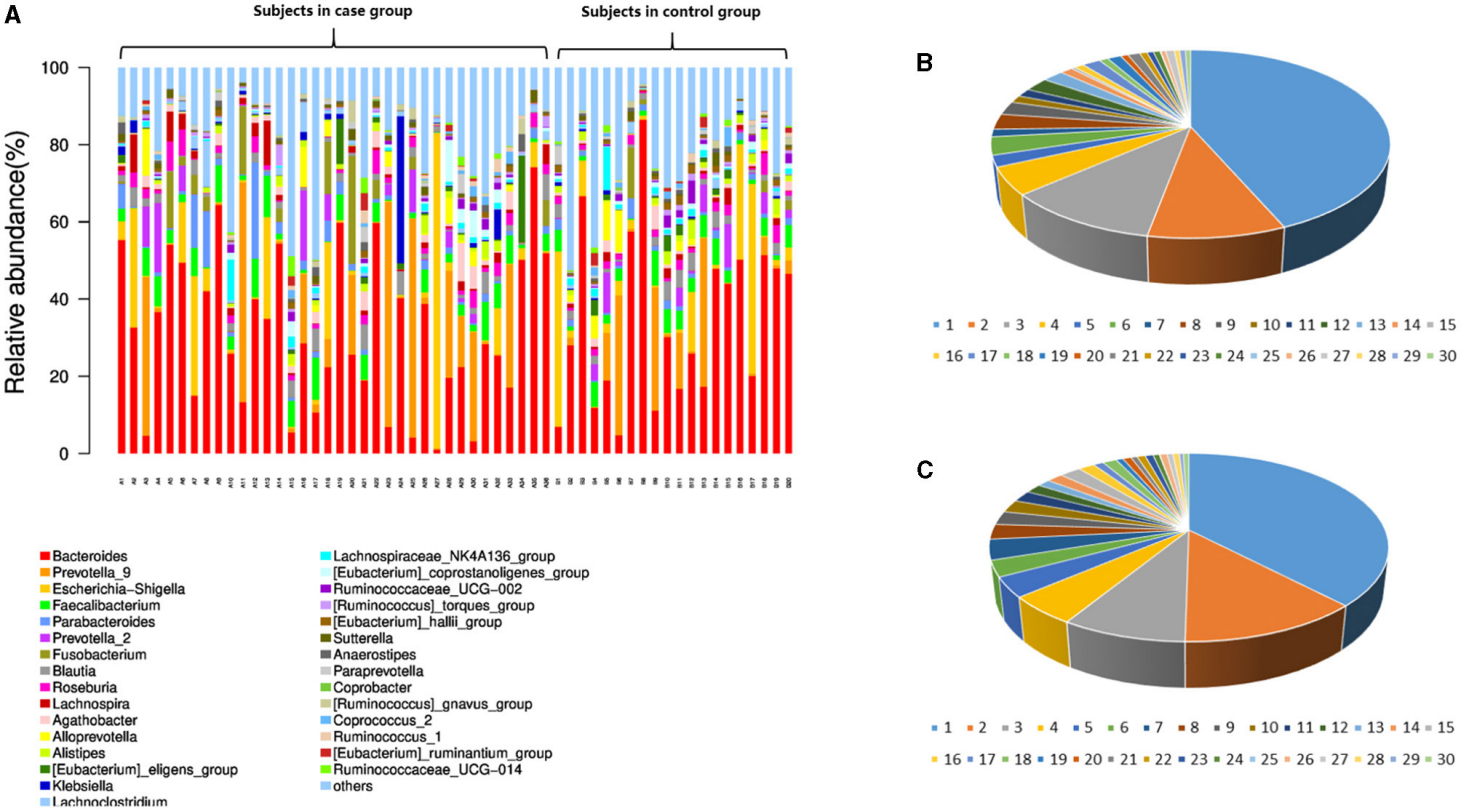
Intestinal microbiota abundance in the species and genus levels in each subject. **(A)** The intestinal microbiota abundance in the genus level; A1-A36 are the participants in the case group. B1-B20 are the participants in the control group. **(B)** The genus abundance in the control group; **(C)** the genus abundance in the case group. In **(B, C)**, the numbers from 1 to 30 are *Bacteroides, Prevotella*_9, *Escherichia Shigella, Faecalibacterium, Parabacteroides, Prevotella*_2, *Fusobacterium, Blautia, Roseburia, Lachnospira, Agathobacter, Alloprevotella, Alistipes, Eubacterium*_*eligens*_group, *Klebsiella, Lachnoclostridium, Lachnospiraceae*_NK4A136_group, *Eubacterium*_*coprostanoligenes*_group, *Ruminococcaceae*_UCG002, *Ruminococcus*_*torques*_group, *Eubacterium*_*hallii*_group, *Sutterella, Anaerostipes, Paraprevotella, Coprobacter, Ruminococcus*_*gnavus*_group, *Coprococcus*_2, *Ruminococcus*_1, *Eubacterium*_*ruminantium*_group, and *Ruminococcaceae*_UCG014.

In the level of the genus from T2D patients, the top 30 genera in order were *Bacteroides, Prevotella*_9, *Escherichia Shigella, Faecalibacterium, Parabacteroides, Prevotella*_2, *Fusobacterium, Blautia, Roseburia, Lachnospira, Agathobacter, Alloprevotella, Alistipes, Eubacterium_eligens_*group, *Klebsiella, Lachnoclostridium, Lachnospiraceae*_NK4A136_group, *Eubacterium*_coprostanoligenes_group, *Ruminococcaceae*_UCG002, *Ruminococcus*_*torques*_group, *Eubacterium*_*hallii*_group, *Sutterella, Anaerostipes, Paraprevotella, Coprobacter, Ruminococcus*_*gnavus*_group, *Coprococcus*_2, *Ruminococcus*_1, *Eubacterium*_ruminantium_group, and *Ruminococcaceae*_UCG014 (Table 2; Figure 2).

**Table 2:** The abundance of the intestinal microbiota in the genus level.

**Genus**	**Case group (*N* = 36)**	**Control group (*N* = 20)**	***p*-value**
*Bacteroides*	0.315327 ± 0.198983	0.344685 ± 0.221181	0.732
*Prevotella*_9	0.103241 ± 0.179453	0.074228 ± 0.128138	0.864
*Escherichia Shigella*	0.070851 ± 0.156658	0.084209 ± 0.150433	0.274
*Faecalibacterium*	0.041341 ± 0.035499	0.037175 ± 0.025136	0.851
*Parabacteroides*	0.028563 ± 0.049757	0.015273 ± 0.010785	0.878
*Prevotella*_2	0.02299 ± 0.048194	0.024469 ± 0.035825	0.081
*Fusobacterium*	0.02919 ± 0.061321	0.010304 ± 0.029492	0.321
*Blautia*	0.021524 ± 0.019495	0.021226 ± 0.014598	0.669
*Roseburia*	0.019633 ± 0.019714	0.018582 ± 0.01335	0.561
*Lachnospira*	0.018314 ± 0.027304	0.010157 ± 0.010689	0.632
*Agathobacter*	0.016382 ± 0.021192	0.011269 ± 0.014041	0.918
*Alloprevotella*	0.013176 ± 0.02866	0.018841 ± 0.032437	0.252
*Alistipes*	0.010478 ± 0.012114	0.016288 ± 0.013625	0.084
*Eubacterium_eligens*_group	0.013046 ± 0.041345	0.00966 ± 0.013258	0.104
*Klebsiella*	0.017171 ± 0.063921	0.002943 ± 0.003094	0.573
*Lachnoclostridium*	0.01318 ± 0.022305	0.007348 ± 0.007942	0.161
*Lachnospiraceae*_NK4A136_group	0.008224 ± 0.017948	0.013905 ± 0.023783	0.040
*Eubacterium_coprostanoligenes*_group	0.011223 ± 0.022309	0.006848 ± 0.005858	0.194
*Ruminococcaceae*_UCG002	0.005574 ± 0.008294	0.010462 ± 0.014852	0.049
*Ruminococcus*_*torques*_group	0.006803 ± 0.009604	0.005712 ± 0.005138	0.891
*Eubacterium*_*hallii*_group	0.004869 ± 0.005232	0.009201 ± 0.007812	0.027
*Sutterella*	0.006695 ± 0.008278	0.005674 ± 0.008579	0.837
*Anaerostipes*	0.006419 ± 0.009887	0.004953 ± 0.004112	0.452
*Paraprevotella*	0.005001 ± 0.011255	0.005084 ± 0.010762	0.412
*Coprobacter*	0.00038 ± 0.000817	0.000492 ± 0.001494	0.485
*Ruminococcus*_*gnavus*_group	0.005925 ± 0.011814	0.003832 ± 0.006472	0.657
*Coprococcus*_2	0.004665 ± 0.008184	0.006709 ± 0.011794	0.128
*Ruminococcus*_1	0.005243 ± 0.00826	0.003948 ± 0.005624	0.720
*Eubacterium*_*ruminantium*_group	0.003096 ± 0.008749	0.004507 ± 0.009361	0.305
*Ruminococcaceae*_UCG014	0.004518 ± 0.011261	0.003965 ± 0.005657	0.116

Among them, the genus levels of *Lachnospiraceae*_NK4A136_group (0.822 ± 1.795% vs. 1.390 ± 2.378%, *P* < 0.05), *Ruminococcaceae*_UCG002 (0.557 ± 0.829% vs. 1.046% ± 1.485%, *P* < 0.05), and *Eubacterium_hallii*_group (0.487 ± 0.523% vs. 0.920 ± 0.781%, *P* < 0.05) were lower in T2D patients than in controls.

**Figure 3:**
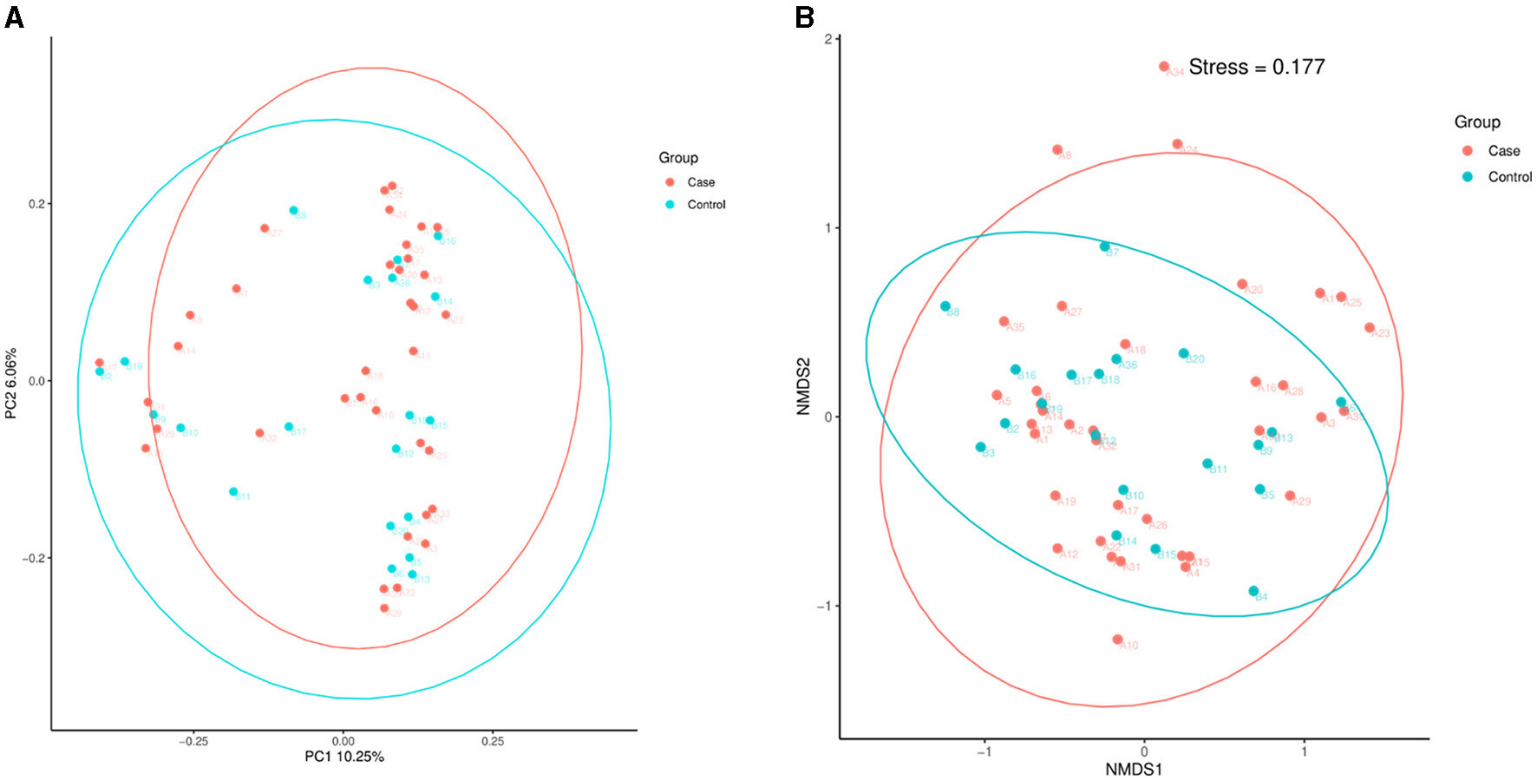
PCoA and NMDS. **(A)** PCoA. **(B)** NMDS analysis. PCoA, principal coordinate analysis; NMDS, non-metric multidimensional scaling; T2D, type 2 diabetes.

As Figure 3 illustrates, the PCoA and NMDS analyses showed that the beta diversity of the intestinal microflora was not significantly different between the case and control groups.

**Figure 4:**
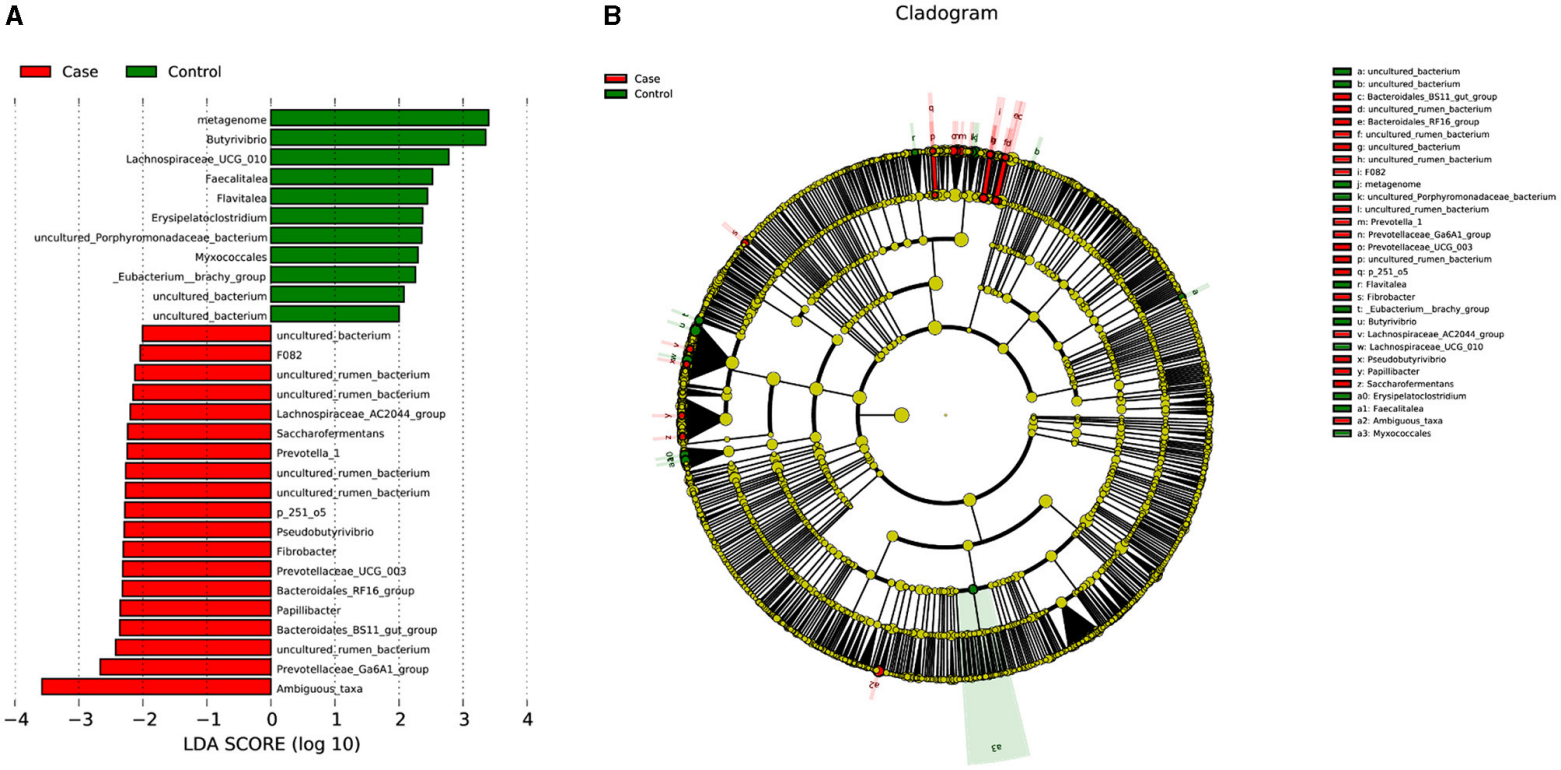
LDA score and cladogram. **(A)** LDA score. **(B)** Cladogram. LDA, linear discriminant analysis.

Linear discriminant analysis (LDA) was used to determine the effect size (LEfSe) of each distinct flora. At an LDA value of 2, and a *p*-value of < 0.05 after correcting the false discovery rate (FDR), fecal samples from the case group exhibited 19 microorganisms, which included Bacteria.Firmicutes.Clostridia.Clostridiales.Lachnospiraceae.Lachnospiraceae_AC2044_group, Bacteria.Firmicutes.Clostridia.Clostridiales.Lachnospiraceae.Pseudobutyrivibrio, Bacteria.Bacteroidetes.Bacteroidia .Bacteroidales.Bacteroidales_RF16_group.uncultured_rumen_bacterium, Bacteria.Bacteroidetes.Bacteroidia .Bacteroidales.Bacteroidales_RF16_group, Bacteria.Bacteroidetes.Bacteroidia .Bacteroidales.p_251_o5.uncultured_rumen_bacterium, Bacteria.Bacteroidetes.Bacteroidia .Bacteroidales.Prevotellaceae.Prevotellaceae_UCG_003, Bacteria.Bacteroidetes.Bacteroidia .Bacteroidales.Prevotellaceae.Prevovtellav_1, Bacteria.Bacteroidetes.Bacteroidia .Bacteroidales.F082.uncultured_rumen_bacterium, Bacteria.Fibrobacteres.Fibrovbacteria.Fibrobacterales.Fibrobacvteraceae.Fibrobvacter, Bacteria.Bacteroidetes.Bacteroidia .Bacteroidales.Prevotellaceae.Prevotvellaceae_Ga6A1_group, Bacteria.Bacteroidetes.Bacteroidia .Bacteroidales.F082, Bacteria.Bacteroidetes.Bacteroidia .Bacteroidales.Bacteroidales_BS11_gut_group, Bacteria.Bacteroidetes.Bacteroidia .Bacteroidales.p_251_o5, Bacteria.Firmicutes.Clostridia.Clostridiales.Ruminococcaceae.Saccharofermentans, Bacteria.Bacteroidetes.Bacteroidia .Bacteroidales.Muribaculaceae.uncultured_rumen_bacterium, Bacteria.Firmicutes.Clostridia.Clostridiales.Ruminococcaceae.Papillibacter, Bacteria.Bacteroidetes.Bacteroidia .Bacteroidales.Bacteroidales_UCG_001.uncultured_rumen_bacterium, Bacteria.Bacteroidetes.Bacteroidia .Bacteroidales.F082.uncultured_bacterium, and Bacteria.Proteobacteria.Alphaproteobacteria.Rhodospirillales.uncultured.Ambiguous_taxa (Figure 4; Table 3). In contrast, 11 microorganisms from the control group were found, which included Bacteria.Firmicutes.Erysipelotrichia.Erysipelotrichales.Erysipelotrichaceae.Faecalitalea, Bacteria.Actinobacteria.Thermoleophilia.Gaiellales.uncultured.uncultured_bacterium, Bacteria.Bacteroidetes.Bacteroidia.Bacteroidales.Muribaculaceae.metagenome, Bacteria.Firmicutes.Clostridia.Clostridiales.Family_XIII._Eubacterium__brachy_group, Bacteria.Proteobacteria.Deltaproteobacteria.Myxococcales, Bacteria.Bacteroidetes.Bacteroidia .Bacteroidales.Muribaculaceae.uncultured_Porphyromonadaceae_bacterium, Bacteria.Firmicutes.Erysipelotrichia.Erysipelotrichales.Erysipelotrichaceae.Erysipelatoclostridium, Bacteria.Firmicutes.Clostridia.Clostridiales.Lachnospiraceae.Butyrivibrio, Bacteria.Actinobacteria.Acidimicrobiia.Actinomarinales.uncultured.uncultured_bacterium, Bacteria.Firmicutes.Clostridia.Clostridiales.Lachnospiraceae.Lachnospiraceae_UCG_010, and Bacteria.Bacteroidetes.Bacteroidia.Chitinophagales.Chitinophagaceae.Flavitalea (Figure 4; Table 3).

**Table 3:** Biomarkers from the linear discriminant analysis effect size (LEfSe) method.

**Biomarker**	**Logarithm value**	**Groups**	**LDA_value**	***p*-value**
Bacteria.Firmicutes.Clostridia.Clostridiales.Lachnospiraceae.Lachnospiraceae_AC2044_group	1.80971997	Case	2.198728016	0.000783885
Bacteria.Firmicutes.Clostridia.Clostridiales.Lachnospiraceae.Pseudobutyrivibrio	1.66353611	Case	2.290879412	0.014914114
Bacteria.Bacteroidetes.Bacteroidia.Bacteroidales.Bacteroidales_RF16_group.uncultured_rumen_bacterium	1.73150381	Case	2.270600832	0.003120021
Bacteria.Bacteroidetes.Bacteroidia.Bacteroidales.Bacteroidales_RF16_group	1.92689419	Case	2.319573034	0.000514091
Bacteria.Bacteroidetes.Bacteroidia.Bacteroidales.p_251_o5.uncultured_rumen_bacterium	1.26181714	Case	2.268957858	0.015724376
Bacteria.Bacteroidetes.Bacteroidia.Bacteroidales.Prevotellaceae.Prevotellaceae_UCG_003	2.72816214	Case	2.314005599	0.002071552
Bacteria.Bacteroidetes.Bacteroidia.Bacteroidales.Prevotellaceae.Prevotella_1	2.64292538	Case	2.243908008	1.08E-06
Bacteria.Bacteroidetes.Bacteroidia.Bacteroidales.F082.uncultured_rumen_bacterium	1.79941535	Case	2.12516752	0.045119815
Bacteria.Fibrobacteres.Fibrobacteria.Fibrobacterales.Fibrobacteraceae.Fibrobacter	2.05062364	Case	2.305882108	8.39E-06
Bacteria.Bacteroidetes.Bacteroidia.Bacteroidales.Prevotellaceae.Prevotellaceae_Ga6A1_group	2.94674683	Case	2.666578708	0.014677059
Bacteria.Bacteroidetes.Bacteroidia.Bacteroidales.F082	2.36110556	Case	2.041603193	4.30E-05
Bacteria.Bacteroidetes.Bacteroidia.Bacteroidales.Bacteroidales_BS11_gut_group	1.43247509	Case	2.360212808	0.001183106
Bacteria.Bacteroidetes.Bacteroidia.Bacteroidales.p_251_o5	1.26181714	Case	2.281705577	0.015724376
Bacteria.Firmicutes.Clostridia.Clostridiales.Ruminococcaceae.Saccharofermentans	1.68596355	Case	2.237288842	0.000102425
Bacteria.Bacteroidetes.Bacteroidia.Bacteroidales.Muribaculaceae.uncultured_rumen_bacterium	1.44039827	Case	2.42532688	0.012772354
Bacteria.Firmicutes.Clostridia.Clostridiales.Ruminococcaceae.Papillibacter	1.63720269	Case	2.353490318	0.039202102
Bacteria.Bacteroidetes.Bacteroidia.Bacteroidales.Bacteroidales_UCG_001.uncultured_rumen_bacterium	1.72870484	Case	2.153057212	0.040027281
Bacteria.Bacteroidetes.Bacteroidia.Bacteroidales.F082.uncultured_bacterium	2.20397056	Case	2.005412809	4.22E-05
Bacteria.Proteobacteria.Alphaproteobacteria.Rhodospirillales.uncultured.Ambiguous_taxa	3.81185769	Case	3.576465466	0.029687731
Bacteria.Firmicutes.Erysipelotrichia.Erysipelotrichales.Erysipelotrichaceae.Faecalitalea	2.83345128	Control	2.523501758	0.002663119
Bacteria.Actinobacteria.Thermoleophilia.Gaiellales.uncultured.uncultured_bacterium	1.98517274	Control	2.00215955	0.027803861
Bacteria.Bacteroidetes.Bacteroidia.Bacteroidales.Muribaculaceae.metagenome	3.99763486	Control	3.400449734	0.043612938
Bacteria.Firmicutes.Clostridia.Clostridiales.Family_XIII._Eubacterium__brachy_group	2.46540229	Control	2.257036475	0.024454704
Bacteria.Proteobacteria.Deltaproteobacteria.Myxococcales	2.75610065	Control	2.295596705	0.023950387
Bacteria.Bacteroidetes.Bacteroidia.Bacteroidales.Muribaculaceae.uncultured_Porphyromonadaceae_bacterium	2.41333272	Control	2.357584729	0.016373402
Bacteria.Firmicutes.Erysipelotrichia.Erysipelotrichales.Erysipelotrichaceae.Erysipelatoclostridium	2.74794817	Control	2.371019539	0.042442785
Bacteria.Firmicutes.Clostridia.Clostridiales.Lachnospiraceae.Butyrivibrio	3.75970144	Control	3.354054161	0.006171619
Bacteria.Actinobacteria.Acidimicrobiia.Actinomarinales.uncultured.uncultured_bacterium	1.86839059	Control	2.076755462	0.01318685
Bacteria.Firmicutes.Clostridia.Clostridiales.Lachnospiraceae.Lachnospiraceae_UCG_010	3.27785299	Control	2.778166995	0.010839023
Bacteria.Bacteroidetes.Bacteroidia.Chitinophagales.Chitinophagaceae.Flavitalea	1.49555245	Control	2.44557861	0.003300746

LDA, linear discriminant analysis.

**Figure 5:**
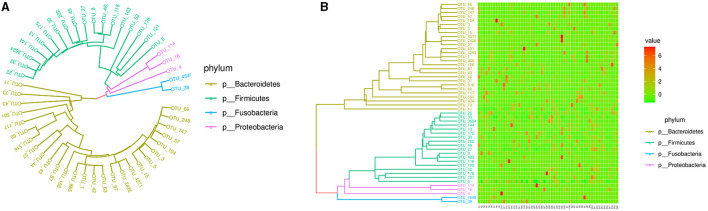
Phylogenetic tree and heatmap of the intestinal microbiota in the top 50. **(A)** Phylogenetic tree. **(B)** Heatmap.

The PICRUSt (Figure 5) did not show the special functional potential of the microbial communities or identify the metabolic pathways based on the KEGG database.

### 3.4 Correlations

**Figure 6:**
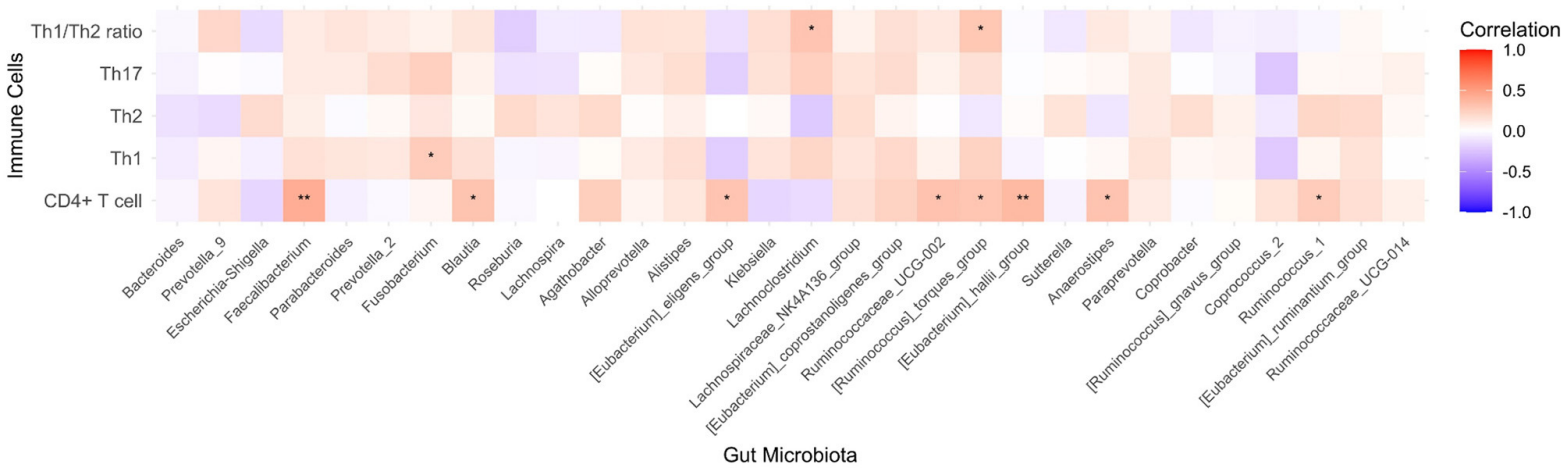
Heatmap of the correlations between the CD4+T subtypes percentage and genus abundance. *p < 0.05, **p < 0.01.

In the level of the genus, when all of the participants were considered together, the CD4+ T cell percentage was positively correlated with the abundance of *Faecalibacterium* (*R* = 0.420, *p* = 0.001), *Blautia* (*R* = 0.306, *p* = 0.022), *Eubacterium*_*eligens*_group (*R* = 0.302, *p* = 0.024), *Ruminococcaceae*_UCG002 (*R* = 0.316, *p* = 0.018), *Ruminococcus*_*torques*_group (*R* = 0.296, *p* = 0.027), *Eubacterium*_*hallii*_group (*R* = 0.361, *p* = 0.006), and *Anaerostipes* (*R* = 0.314, *p* = 0.018). The Th1/Th2 ratio was positively correlated with the abundance of *Lachnoclostridium* (*R* = 0.301, *p* = 0.024) and *Ruminococcus*_*torques*_group (*R* = 0.285, *p* = 0.033). Figure 6 illustrates the correlation between the CD4+T subtype percentage and genus abundance.

### 3.5 Interactions between microbial taxa and Th cell percentages

The network analysis by constructing a co-occurrence network showed that the density of the network was 0.273, the average length of the pathway was 0.850, and the cluster coefficient was 0.625. These results suggested that there is a tight network with medium density, and it may include several small groups *with a close connection*.

**Figure 7:**
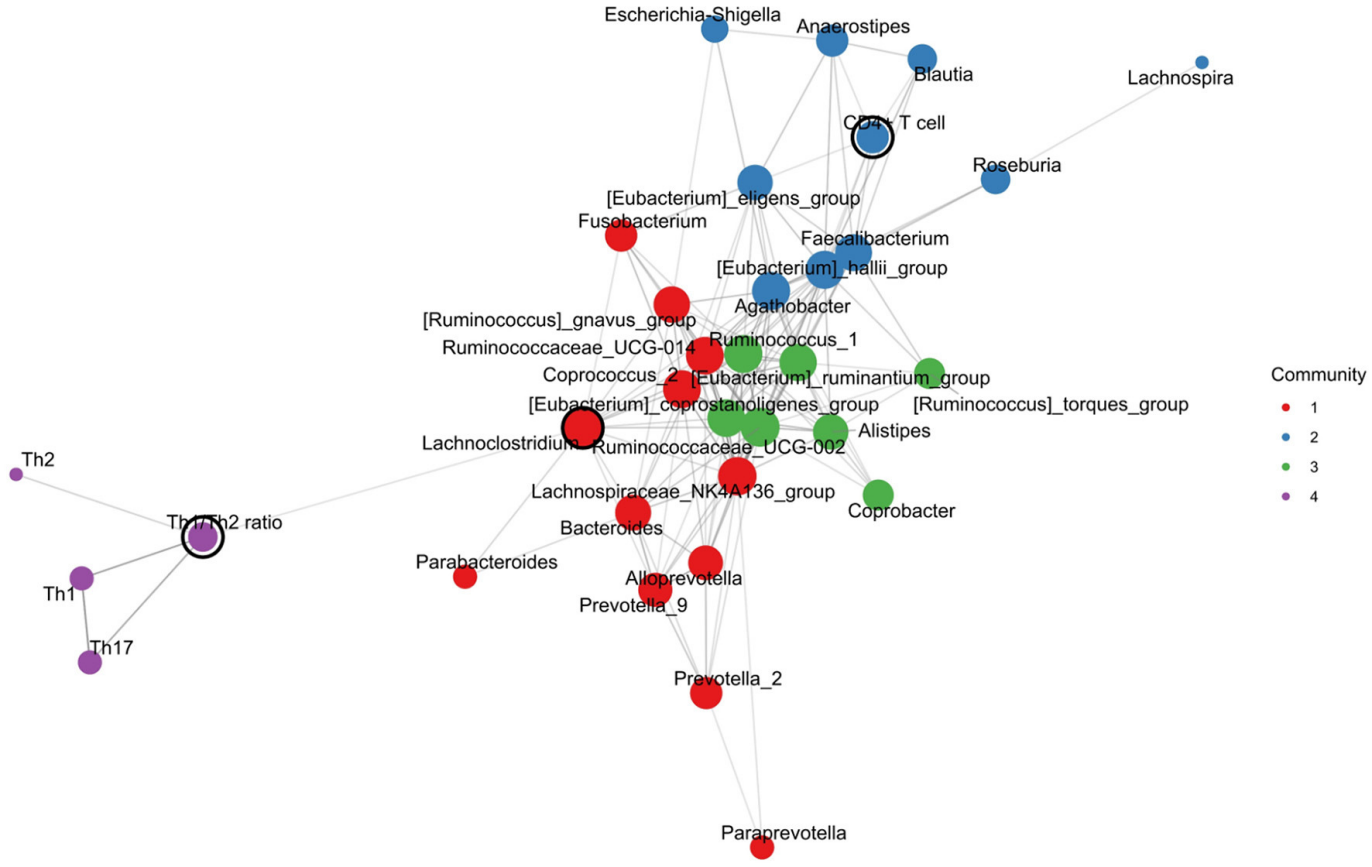
Intestinal microbial-immune cell co-occurrence network. Node size, degree centrality; edge width, association strength; node color, community.

The co-occurrence network showed the top 10 nodes, which were as follows: the *Lachnoclostridium*, Th1/Th2 ratio, *Ruminococcaceae*_UCG-002, *Faecalibacterium, Agathobacter, Lachnospiraceae*_NK4A136_group, *Ruminococcus*_*gnavus*_group, *Roseburia, Bacteroides*, and *Ruminococcus*_1. As shown in Figure 7, four communities were found as follows: Community 1 included the genera *Bacteroides, Prevotella*, and others; Community 2 included some probiotics (i.e., *Faecalibacterium, Blautia*, and *Roseburia*) and CD4+ T cells; Community 3 included several anaerobic bacteria and butyrate-producing bacteria; and Community 4 included the Th1, Th2, Th17, and Th1/Th2 ratios.

## 4 Discussion

In this study, the percentages of Th1 and Th17 in the peripheral blood were higher in patients with T2D than in controls. Among the top 30 genera of the intestinal microbiota, the levels of *Lachnospiraceae*_NK4A136_group, *Ruminococcaceae*_UCG002, and *Eubacterium*_*hallii*_group were lower in patients with T2D than in controls. Moreover, the Th1/Th2 ratio was positively correlated with the abundance of the *Lachnoclostridium* and *Ruminococcus*_*torques*_group genera.

In our study, the genera of *Ruminococcaceae*_UCG002, *Lachnospiraceae*_NK4A136_group, and *Eubacterium*_*hallii*_group were decreased in patients with T2D compared with controls. The LEfSe analysis also showed that the *Lachnospiraceae* and *Ruminococcaceae* families were significantly different between T2D participants and controls. In previous studies, these findings were not reported in T2D patients. In a study, unknown *Ruminococcaceae* was found to be decreased in diabetic cats compared to healthy and lean cats, which was negatively correlated with the levels of serum amyloid A (Kieler et al., 2019). Our study identified a decreased *Ruminococcaceae*_UCG002 in T2D patients. Moreover, in another study, *Lachnospiraceae*_UCG-004 was increased in Chinese patients with T2D with stage III diabetic nephropathy (Du et al., 2021). In contrast, the present study found decreased *Lachnospiraceae*_NK4A136_group genus in T2D patients without diabetic nephropathy. These findings suggested that diabetic complications related to the intestinal microbiota ecology in the genus level. In addition, *Eubacterium*_*hallii* is an anaerobic bacterium belonging to the butyrate-producing *Lachnospiraceae* family. The treatment to increase *Eubacterium_hallii* in the intestinal microbiota could improve insulin sensitivity in a mice model and in T2D patients (Udayappan et al., 2016; Estrella et al., 2024). Our findings support that the *Eubacterium*_*hallii* supplement could be effective for the treatment of T2D, but clinical trials are needed to confirm it in the future.

In the present study, the percentages of Th1 and Th17 in the peripheral blood were higher in T2D patients than in controls. Our findings were consistent with previous reports (Sun et al., 2024; Zhang et al., 2021; Zeng et al., 2012). It is widely accepted that Th1 and Th17 are involved in chronic inflammation in T2D (García-Macedo and de los Ángeles Fortis, 2023). In our study, the Th1/Th2 ratio was positively correlated with the abundance of the *Lachnoclostridium* and *Ruminococcus*_*torques*_group genera. In our study, the correlation coefficients were lower, which might have been partly due to the small sample size. The correlation coefficients may be more significant when the sample size is enlarged in future studies. A recent study found that the genus *Lachnoclostridium* retained a significant positive correlation with T2DM risk (Song et al., 2024), which is involved in the pathways of linoleic acid metabolism, serotonergic synapse, and tryptophan metabolism (Li et al., 2023). Moreover, the genus *Ruminococcus*_*torques* is positively associated with impaired fasting glucose (Nogal et al., 2023). It could participate in the activation of the colonic toll-like receptor 4 (TLR4)/nuclear factor kappa-B (NF-κB) pathway, in the downregulation of intestinal tight junction protein expression, and in the promotion of impaired glucose and serum lipid metabolism (Zhao et al., 2023).

Furthermore, we performed a network analysis by constructing a co-occurrence network. In our results, the Th1/Th2 ratio, *Ruminococcaceae*_UCG-002, and *Lachnospiraceae*_NK4A136_group were important nodes of the top 10 nodes, which suggested that these genera may play a central role between the intestinal microbiota and Th cells-related immune response in T2D. Moreover, the CD4+T cell was in the same community with probiotics (i.e., *Faecalibacterium, Blautia*, and *Roseburia*), suggesting a potential interaction between them. These findings were consistent with those from the correlation analysis. Since it remains unclear how the Th cell interacts with a physiologically complex intestinal microbiome in T2D, our findings may provide a novel clue for further exploring the precise mechanism of the gut microbiota–Th cell axis in T2D pathogenesis.

In our study, the PICRUSt did not identify the metabolic pathways based on KEGG, which may be partly due to the small sample size of our study. When the sample size is large in future explorations, more interesting information may be found. The pathways in the analysis did not include those with the CD4+T cell, Th1, or Th2. The pathway related to the Th17 cell differentiation was not found to be related to the microbiome, which was consistent with the findings from the correlation analysis, where no significant correlation was found between Th17 and the intestinal microbiota.

## 5 Conclusion

In conclusion, this study provids a preliminary picture of the crosstalk between the intestinal microbiome and systematic Th cells in patients with T2D. These findings suggested that the network relationship among the intestinal microbiota, metabolites, and CD4+T lymphocyte immunity was unbalanced in T2D patients, which might have promoted the development of T2D. This presents a therapeutic opportunity to modulate gut immune reaction and then chronic inflammation by manipulating microbiome-specific Th-cell response.

## Data Availability

The data presented in the study are deposited in the Figshare repository: https://figshare.com/articles/dataset/data-intestinal_flora-T2D_xlsx/27069130?file=49302850.
